# Folic Acid Supplementation Improves Glycemic Control for Diabetes Prevention and Management: A Systematic Review and Dose-Response Meta-Analysis of Randomized Controlled Trials

**DOI:** 10.3390/nu13072355

**Published:** 2021-07-09

**Authors:** Omid Asbaghi, Damoon Ashtary-Larky, Reza Bagheri, Seyedeh Parisa Moosavian, Hadi Pourmirzaei Olyaei, Behzad Nazarian, Mahnaz Rezaei Kelishadi, Alexei Wong, Darren G. Candow, Frédéric Dutheil, Katsuhiko Suzuki, Amirmansour Alavi Naeini

**Affiliations:** 1 Cancer Research Center, Shahid Beheshti University of Medical Sciences, Tehran 1416753955, Iran; omid.asbaghi@gmail.com; 2 Nutrition and Metabolic Diseases Research Center, Ahvaz Jundishapur University of Medical Sciences, Ahvaz 6135715794, Iran; damoon_ashtary@yahoo.com; 3 Department of Exercise Physiology, University of Isfahan, Isfahan 8174673441, Iran; will.fivb@yahoo.com; 4 Department of Clinical Nutrition, School of Nutrition and Food Science, Isfahan University of Medical Sciences, Isfahan 8174673461, Iran; p_moosavian@yahoo.com; 5 Department of Nutrition, Science and Research Branch, Islamic Azad University, Tehran 1584743311, Iran; h.pourmirzaei@srbiau.ac.ir; 6 Student Research Committee, Lorestan University of Medical Sciences, Khorramabad 6813833946, Iran; nazarianbehzad969@yahoo.com; 7 Department of Community Nutrition, School of Nutrition and Food Science, Isfahan University of Medical Sciences, Isfahan 8174673461, Iran; m.rezaei81@yahoo.com; 8 Department of Health and Human Performance, Marymount University, Arlington, VA 22207, USA; awong@marymount.edu; 9 Faculty of Kinesiology and Health Studies, University of Regina, Regina, SK S4S OA2, Canada; Darren.Candow@uregina.ca; 10 CNRS, LaPSCo, Physiological and Psychosocial Stress, CHU Clermont-Ferrand, University Hospital of Clermont-Ferrand, Preventive and Occupational Medicine, Université Clermont Auvergne, WittyFit, F-63000 Clermont-Ferrand, France; fred_dutheil@yahoo.fr; 11 Faculty of Sport Sciences, Waseda University, 2-579-15 Mikajima, Tokorozawa 359-1192, Japan

**Keywords:** folic acid, glycemic control, diabetes, meta-analysis

## Abstract

Background: There is a growing interest in the considerable benefits of dietary supplementations, such as folic acid, on the glycemic profile. We aimed to investigate the effects of folic acid supplementation on glycemic control markers in adults. Methods: Randomized controlled trials examining the effects of folic acid supplementation on glycemic control markers published up to March 2021 were detected by searching online databases, including Scopus, PubMed, Embase, and ISI web of science, using a combination of related keywords. Mean change and standard deviation (SD) of the outcome measures were used to estimate the mean difference between the intervention and control groups at follow-up. Meta-regression and non-linear dose-response analysis were conducted to evaluate the association between pooled effect size and folic acid dosage (mg/day) and duration of the intervention (week). From 1814 detected studies, twenty-four studies reported fasting blood glucose (FBG), fasting insulin, hemoglobin A1C (HbA1C), and Homeostatic Model Assessment for Insulin Resistance (HOMA-IR) as an outcome measure. Results: Results revealed significant reductions in FBG (weighted mean difference (WMD): −2.17 mg/dL, 95% CI: −3.69, −0.65, *p* = 0.005), fasting insulin (WMD: −1.63 pmol/L, 95% CI: −2.53, −0.73, *p* < 0.001), and HOMA-IR (WMD: −0.40, 95% CI: −0.70, −0.09, *p* = 0.011) following folic acid supplementation. No significant effect was detected for HbA1C (WMD: −0.27%, 95% CI: −0.73, 0.18, *p* = 0.246). The dose-response analysis showed that folic acid supplementation significantly changed HOMA-IR (r = −1.30, p-nonlinearity = 0.045) in non-linear fashion. However, meta-regression analysis did not indicate a linear relationship between dose, duration, and absolute changes in FBG, HOMA-IR, and fasting insulin concentrations. Conclusions: Folic acid supplementation significantly reduces some markers of glycemic control in adults. These reductions were small, which may limit clinical applications for adults with type II diabetes. Further research is necessary to confirm our findings.

## 1. Introduction

Glycemic management among individuals suffering from chronic metabolic disease seems indispensable in preventing further complications [1]. The global incidence of diabetes mellitus, one of the primary endocrine disorders associated with poor glycemic control, has progressively climbed to 415 million adults; and this number will reach 649 million by 2040 [2]. Type 2 diabetes mellitus (T2DM) is the predominant type of metabolic disease, accounting for 90–95% of cases, which are characterized by high blood glucose, insulin resistance (Homeostatic Model Assessment for Insulin Resistance [HOMA-IR]), and relative lack of insulin [3,4]. To combat T2DM complications, several efficient approaches have been recommended, such as lifestyle modifications, pharmacological treatment, exercise interventions, changes in either dietary intakes, the inclusion of food supplements, or in combination [5,6].

It has been assumed that dietary supplements have led to promising outcomes for T2DM treatment [7,8,9,10]. Namely, folic acid is known as one of the members of the vitamin B complex, which is crucial for red blood cell (RBC) increment and other body cells as an essential vitamin. Folic acid is also recognized as folic acid, folate, vitamin B9, and vitamin B11 [11]. One of the forms of folic acid naturally in food products is “folate”, a water-soluble vitamin [12]. Evidence indicates that folic acid supplementation could positively affect inflammatory and oxidative stress markers [13,14]. However, the reported effects of folic acid on glycemic control have been inconclusive. For instance, folic acid supplementation reduced plasma concentrations of homocysteine and improves glycemic control, insulin resistance, and vitamin B12 in those with T2DM who consume high doses of metformin [15]. In addition, in patients with metabolic syndrome, folate supplementation decreases insulin resistance and improves endothelial dysfunction [16]. According to a few small randomized controlled trials (RCTs), folic acid supplementation also has possible advantages for decreasing insulin resistance [17,18]. On the other hand, some previous studies failed to show any benefit in patients with T2DM. For instance, a study has shown an increase in glucose concentrations caused by a short-term, low-dose folic acid supplementation [19]. Moreover, Mangoni et al. reported that although folic acid supplementation can significantly decrease serum concentrations of homocysteine, there were no significant changes in lipid and glycemic profile following folic acid supplementation in patients with T2DM [20]. Similar outcomes were observed in other studies [21,22].

Some meta-analytic studies have evaluated the efficacy of folic acid supplementation on various glycemic control markers [23,24,25]. However, these meta-analyses either failed to include some relevant studies, did not perform subgroup or dose-response analysis, meta-regression, or had low overall certainty of evidence across the studies included. Collectively, findings on the effects of folic acid supplementation on glycemic control remain unclear, which demonstrates a need for an updated comprehensive systematic review and meta-analysis of RCTs on this topic. Consequently, we performed this systematic review and meta-analysis of published RCTs to summarize available findings on the influence of folic acid supplementation on glycemic control in adults.

## 2. Materials and Methods

This study was performed based on the Preferred Reporting Items for Systematic Reviews and Meta-Analysis (PRISMA) guides [26].

### 2.1. Search Strategy

A systematic search was carried out on electronic databases, including Scopus, PubMed, Embase, and ISI web of science up to March 2021. Consequently, all the studies, which met the inclusion criteria, were added into Endnote software for screening. In our search strategy, we used MeSH terms, keywords, and abstracts, as well as ((“folate”[Title/Abstract] OR “folic acid”[Title/Abstract] OR “Vitamin M”[Title/Abstract] OR “Vitamin B9”[Title/Abstract] OR “Folacin”[Title/Abstract] OR “Folvite”[Title/Abstract] OR “Pteroylglutamic Acid”[Title/Abstract] OR “folates”[Title/Abstract] OR “tetrahydrofolates”[Title/Abstract] OR “Formyltetrahydrofolates”[Title/Abstract]) OR “methylTHF” [Title/Abstract] OR “THF” [Title/Abstract] AND (“glucose tolerance”[Title/Abstract] OR “insulin resistance”[Title/Abstract] OR FBG[Title/Abstract] OR “fasting blood glucose”[Title/Abstract] OR HbA1c[Title/Abstract] OR “hemoglobin A1c”[Title/Abstract] OR HOMA-IR[Title/Abstract] OR “homeostatic model assessment”[Title/Abstract] OR Insulin[Title/Abstract] OR “fasting blood sugar”[Title/Abstract] OR FBS[Title/Abstract])) AND (Intervention[Title/Abstract] OR “Intervention Study”[Title/Abstract] OR “Intervention Studies”[Title/Abstract] OR “controlled trial”[Title/Abstract] OR randomized[Title/Abstract] OR randomized[Title/Abstract] OR random[Title/Abstract] OR randomly[Title/Abstract] OR placebo[Title/Abstract] OR “clinical trial”[Title/Abstract] OR Trial[Title/Abstract] OR “randomized controlled trial”[Title/Abstract] OR “randomized clinical trial”[Title/Abstract] OR RCT[Title/Abstract] OR blinded[Title/Abstract] OR “double blind”[Title/Abstract] OR “double blinded”[Title/Abstract] OR trial[Title/Abstract] OR “clinical trial”[Title/Abstract] OR trials[Title/Abstract] OR “Pragmatic Clinical Trial”[Title/Abstract] OR “Cross-Over Studies”[Title/Abstract] OR “Cross-Over”[Title/Abstract] OR “Cross-Over Study”[Title/Abstract] OR parallel[Title/Abstract] OR “parallel study”[Title/Abstract] OR “parallel trial”[Title/Abstract]). We did not use limitations on time of publication and language. Additionally, unpublished studies and duplicate citations were removed. Two authors (BN and MRK) completed the research process individually and in duplicate. Meeting and consultations with another researcher (OA) solved any disagreements in these regards.

### 2.2. Inclusion Criteria

Two investigators selected qualified studies that conform to the following criteria: studies that evaluated the effect of folic acid supplementation on glycemic control in adult populations (aged > 18 years old), those that reported glycemic control values (fasting blood glucose (FBG), fasting insulin, hemoglobin A1C (HbA1C), homeostatic model assessment for insulin resistance (HOMA-IR)) at baseline and the end of the intervention, and parallel or crossover studies with a control group.

### 2.3. Exclusion Criteria

In vitro studies, animal experiments, case reports, trials without a control group, observational studies, studies using folic acid supplementation in combination with other components, and those investigations that did not report inclusion criteria were excluded from the analysis.

### 2.4. Data Extraction

The following data were extracted from qualified studies by two independent investigators (BN and MRK) using a predetermined abstraction form: (I) first author’s name; (II) publication year; (III) study design and blinding; (IV) duration of the study; (V) characteristics of participants (gender, age, and diseases) in each group; (VI) location of the study; (VII) sample size in control and intervention groups; (VIII) type and dose of placebo and folic acid; and (IX) mean and standard deviation (SD) of outcomes of the glycemic control markers at baseline and the end of the study. When data were published in two different studies, we included the latest one. Moreover, if data for glycemic control markers were reported in various units, we converted to the most commonly used. In cases of the absence of relevant data, we communicated to corresponding authors through e-mail to receive the required information. If there was any inconsistency, that was settled by consensus.

### 2.5. Quality Assessment

To measure the risk of bias for each included study, we used Cochrane collaboration’s tool [27], which contained seven domains: allocation concealment, performance bias, random sequence generation, reporting bias, and detection, attrition bias, and other sources of bias. The quality assessment procedure was described in our earlier studies [8,28]. Moreover, two independent investigators (OA and DAL) performed the risk of bias assessment.

### 2.6. Statistical Analysis

The mean differences and SD for the intervention and control groups were extracted to launch an investigation into the effect size for glycemic control markers. Additionally, weighted mean differences (WMDs) with 95% confidence intervals (CIs) were calculated by a random-effects model. Cochran’s Q test was used to evaluate between-study heterogeneity and I^2^ measurement was used for quantification. I^2^ greater than 40% or *p* < 0.05 was deemed as high between-study heterogeneity [29]. To distinguish probable sources of heterogeneity, we performed a subgroup analysis in conformity with the baseline serum fasting blood glucose concentrations, study duration (<12 and ≥12 weeks), intervention dosage (<5 and ≥5 mg/d), diabetes status (non-T2DM and T2DM), and sex (both sexes, male and female). To conduct sensitivity analysis, we removed each study one after another and recalculated the pooled assessments. To detect potential publication bias, funnel plots and Egger’s regression test were performed. Meta-regression and non-linear dose-response analysis was conducted to evaluate the association between pooled effect size and folic acid dosage (mg/day) and duration of the intervention (week) [30]. Statistical analysis was conducted using STATA, version 11.2 (Stata Corp, College Station, TX, USA). In all analyses, the statistically substantial value was considered as *p* < 0.05.

### 2.7. Certainty Assessment

The overall certainty of evidence across the studies was graded according to the GRADE guidelines (Grading of Recommendations Assessment, Development, and Evaluation) working group. According to the corresponding evaluation criteria, the quality of evidence was classified into four categories: high, moderate, low, and very low [31].

## 3. Results

### 3.1. Study Selection

**Figure 1:**
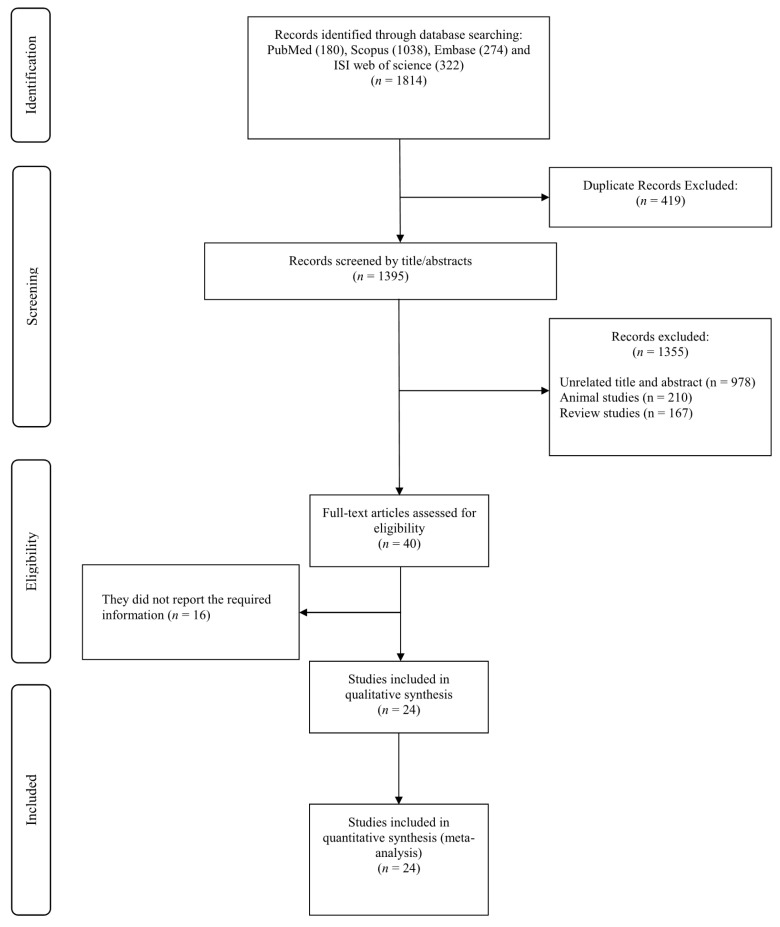
Flowchart of study selection for inclusion studies.

The initial search yielded 1814 studies; however, 419 of those were removed due to duplication. Another 1395 studies were excluded due to unrelated titles and abstracts (*n* = 978), animal studies (*n* = 210), and review studies (*n* = 167). Consequently, 40 relevant studies remained for full-text review. Among these, 16 studies were excluded because of a lack of reporting of glycemic control markers. Finally, 24 studies [15,17,20,21,22,32,33,34,35,36,37,38,39,40,41,42,43,44,45,46,47,48,49,50] were included in the present meta-analysis (Figure 1).

### 3.2. Characteristics of the Included Studies

The characteristics of the included studies are shown in Table 1. In total, 34,646 volunteers were included (case = 17,396; control = 17,249), and the dates of publications were between 1998 and 2018. Twenty-one studies were designed as parallel studies and three as a crossover. The study duration ranged between 3 and 234 weeks, and the sample size ranged from 20 to 20,030 participants. Participants’ ages ranged from 24 to 65 years, while baseline body mass index (BMI) varied between 23.9 and 32.3 kg/m^−2^. Two studies enrolled only males, while nine studies enrolled females. The remained studies enrolled both females and males. Results from quality assessments are indicated in Table 2.

### 3.3. The Effect of Folic Acid Supplementation on FBG

**Figure 2:**
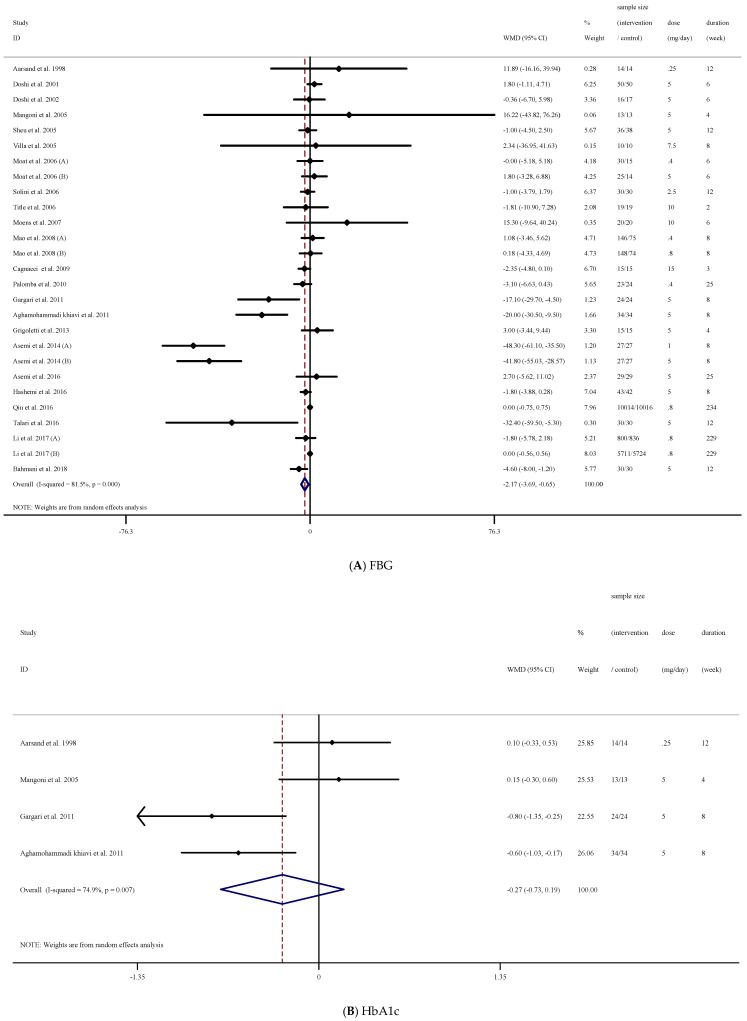

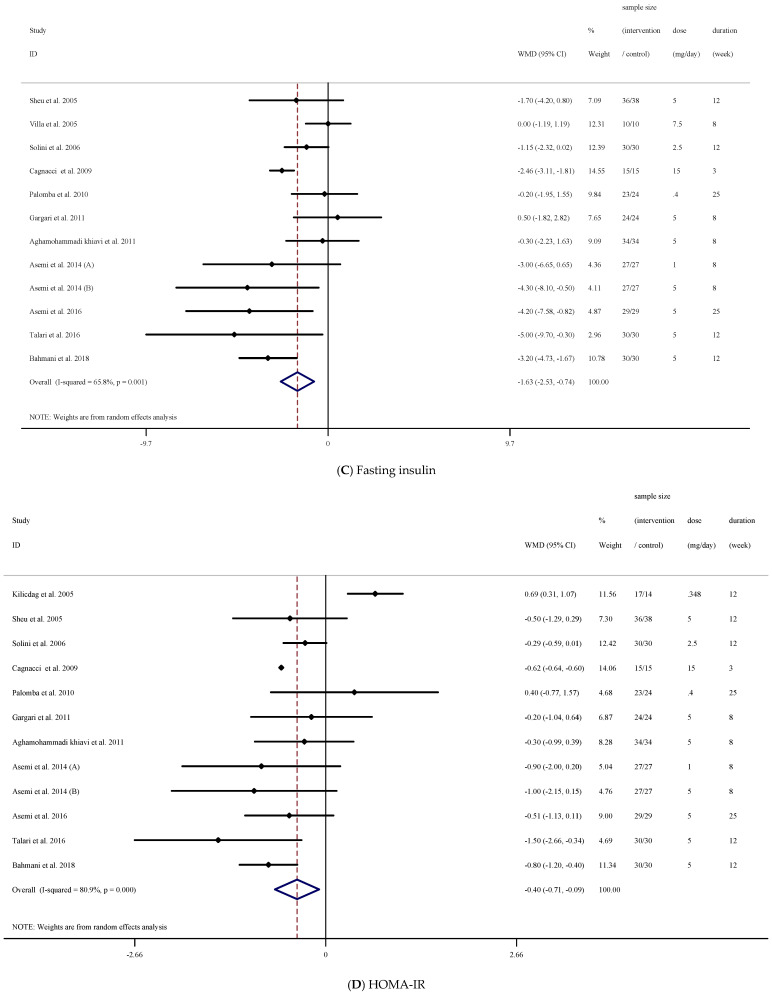
Forest plot detailing weighted mean difference and 95% confidence intervals (CIs) for the effect of acid folic supplementation on; (**A**) FBG; (**B**) HbA1c; (**C**) fasting insulin; (**D**) HOMA-IR.

The results of our analysis demonstrated that folic acid supplementation significantly reduced FBG concentrations ((WMD = −2.17 mg/dL; 95% CI: −3.69, −0.65, *p* = 0.005), (Figure 2A)). However, there was a significant between-study heterogeneity [(I^2^ = 81.5%, *p* < 0.001)]. Therefore, we conducted a subgroup analysis to detect the source of heterogeneity. We found that intervention dose, trial duration, and participant’s sex were the sources of heterogeneity. In addition, subgroup analysis demonstrated that folic acid supplementation significantly reduced FBG concentrations when the study duration was <12 weeks (*p* = 0.006) in participants with higher baseline FBG concentrations (FBG ≥ 100 mg/dL, *p* = 0.043). Moreover, when the dosage was ≥5 (mg/d), a significant decrease in FBG concentrations was detected (*p* = 0.021). These decreases were significant in non-diabetic participants (*p* = 0.030). Lastly, in those studies that enrolled both males (*p* < 0.001) and females (*p* = 0.001) with elevated FBG, a significant reduction in FBG concentrations was found ((*p* = 0.043); (Table 3)).

### 3.4. The Effect of Folic Acid Supplementation on HbA1c

Overall analysis of the data from four effect sizes demonstrated that folic acid supplementation did not significantly affect HbA1c ((WMD = −0.27%; 95% CI: −0.73, 0.18, *p* = 0.246), (Figure 2B)). However, significant heterogeneity existed (I^2^ = 74.9%, *p* = 0.007). In addition, due to the low number of effect sizes, we could not conduct subgroup analysis (Table 3).

### 3.5. The Effect of Folic Acid Supplementation on Fasting Insulin

There was a significant reduction in fasting insulin concentrations following folic acid supplementation ((WMD = −1.63 uU/mL; 95% CI: −2.53, −0.73, *p* < 0.001), (Figure 2C)) with significant between-study heterogeneity ((I^2^ = 65.8%, *p* = 0.001)). The reduction was significant when study duration was ≥12 weeks (*p* = 0.002). In addition, the effects of folic acid supplementation at dosages <5 (*p* = 0.040) and ≥5 ((mg/d); *p* = 0.001)) efficaciously reduced fasting insulin concentrations that were statistically significant in non-T2M patients (*p* < 0.001). Astonishingly, folic acid supplementation favorably attenuated fasting insulin concentrations in females (*p* < 0.001) but not in men ((*p* = 0.972); (Table 3)).

### 3.6. The Effect of Folic Acid Supplementation on HOMA-IR

A significant reduction for HOMA-IR ((WMD = −0.40; 95% CI: −0.70, −0.09, *p* = 0.011), (Figure 2D)) was found following folic acid supplementation. In addition, when supplementation duration was <12 weeks, a significant decrease in HOMA-IR was found (*p* < 0.001). Moreover, doses ≥5 mg/d significantly reduced HOMA-IR (*p* < 0.001). These decrements were significant in non-T2DM patients (*p* = 0.016); (Table 3)).

### 3.7. Publication Bias

**Figure 3:**
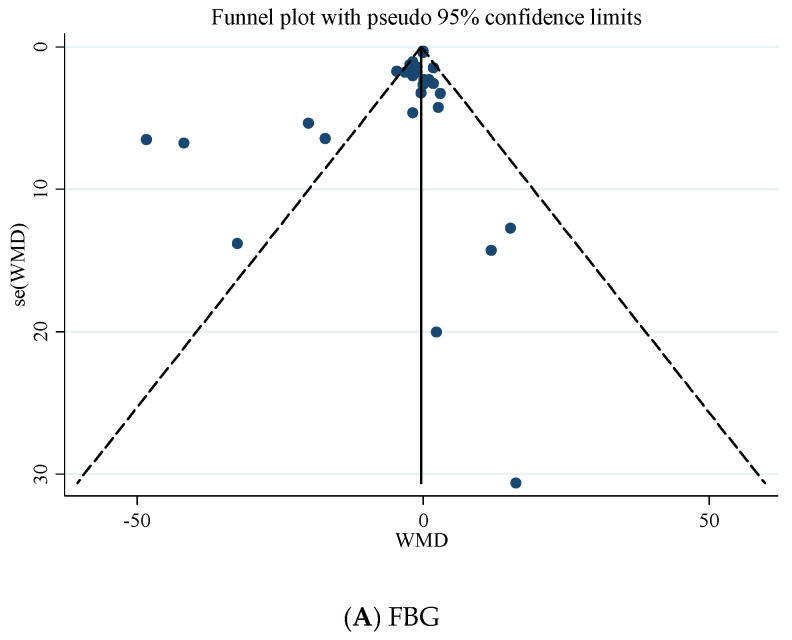

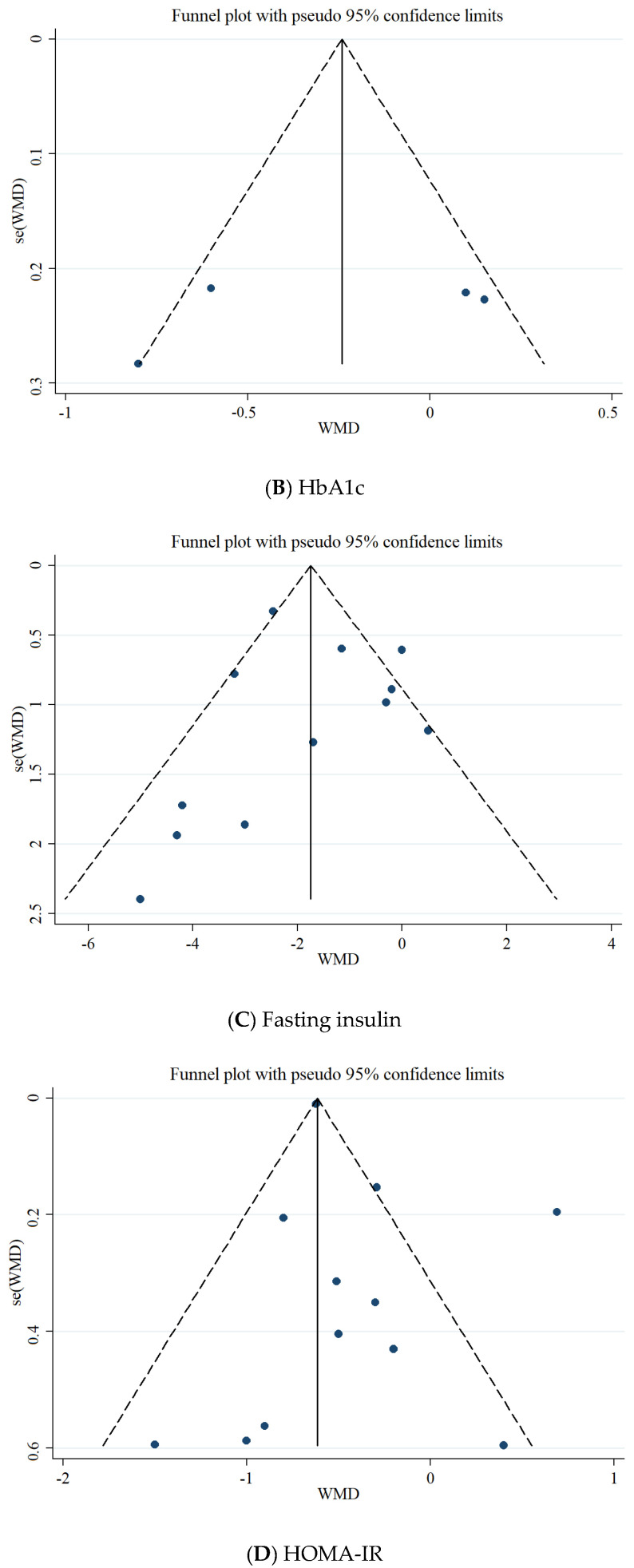
Funnel plot for the effect of folic acid supplementation on; (**A**) FBG; (**B**) HbA1c; (**C**) fasting insulin; (**D**) HOMA-IR.

Based on Egger’s regression test, there was no evidence of publication bias for studies examining the effects of folic acid supplementation on HbA1c (*p* = 0.485), fasting insulin concentrations (*p* = 0.964), and HOMA-IR (*p* = 0.240). However, based on the Egger regression test, there was a significant publication bias for FBG (*p* = 0.028). Additionally, Begg’s test revealed that there is no publication bias for FBG (*p* = 0.588), HbA1c (*p* = 1.000), fasting insulin (*p* = 0.537), and HOMA-IR (*p* = 0.732). The visually inspected funnel plot test also confirmed this point (Figure 3A–D).

### 3.8. Grade Assessment

Grade assessment demonstrated very low quality for FBG concentrations due to serious limitations regarding indirectness, publication bias, and very serious limitations about inconsistency. HbA1c very low because of serious limitation for inconsistency, indirectness and imprecision. Fasting insulin and HOMA-IR had low quality of evidence due to inconsistency, and indirectness (Table 4).

### 3.9. Sensitivity Analysis

Sensitivity analysis for FBG, HbA1c, and fasting insulin concentrations did not show evidence of sensitivity; however, sensitivity analysis for HOMA-IR demonstrated that the overall effect was influenced by the elimination of a study conducted by Aghamohammadi khiavi et al. [42] (WMD = −0.15; 95% CI: −0.71, 0.39).

### 3.10. Non-Linear Dose-Response between Dose and Duration of Folic Acid Supplementation and Glycemic Control

**Figure 4:**
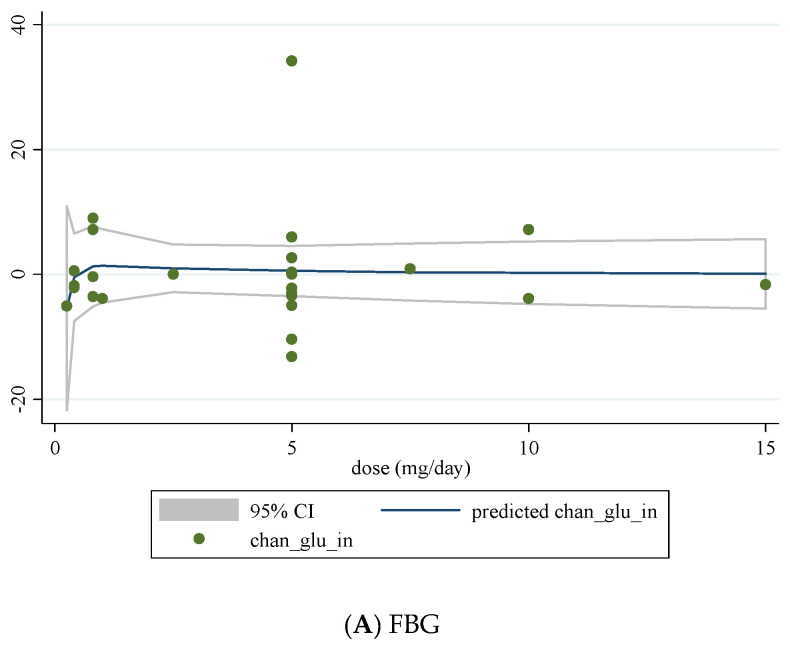

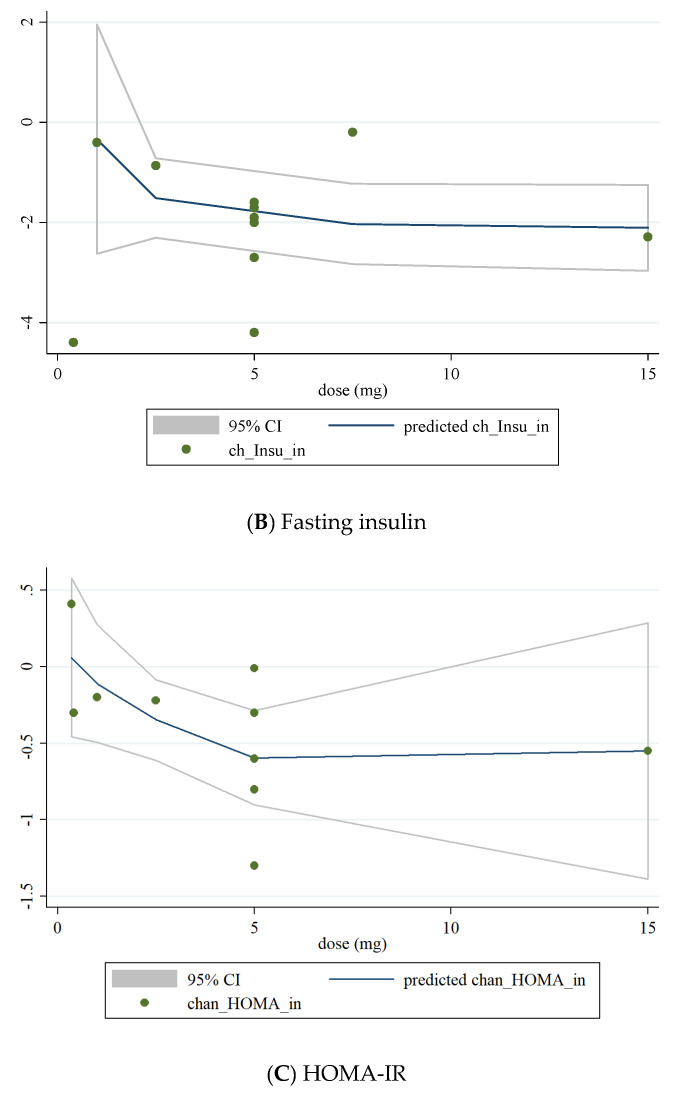
Non-linear dose-response relations between dose of folic acid supplementation (mg/day) and absolute mean differences in (**A**) FBG; (**B**) fasting insulin; (**C**) HOMA-IR.

**Figure 5:**
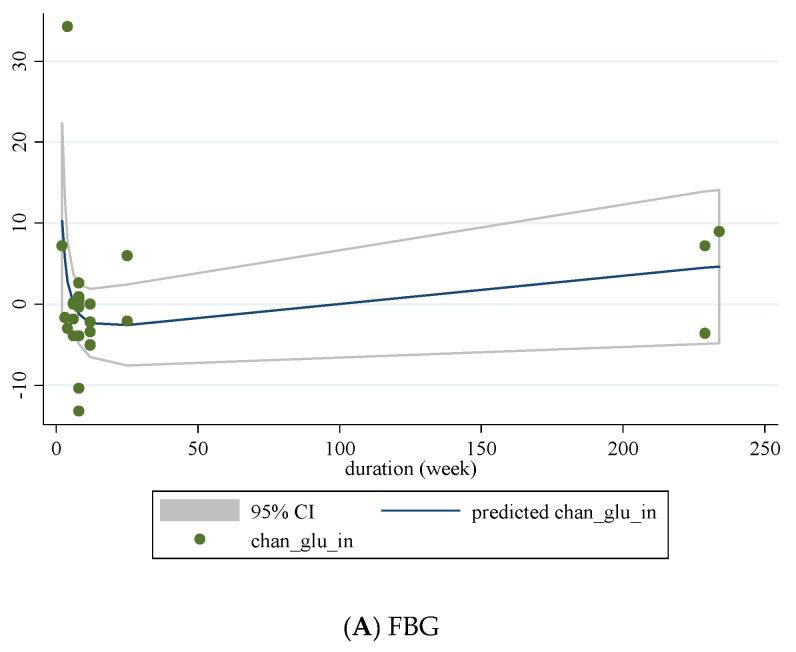

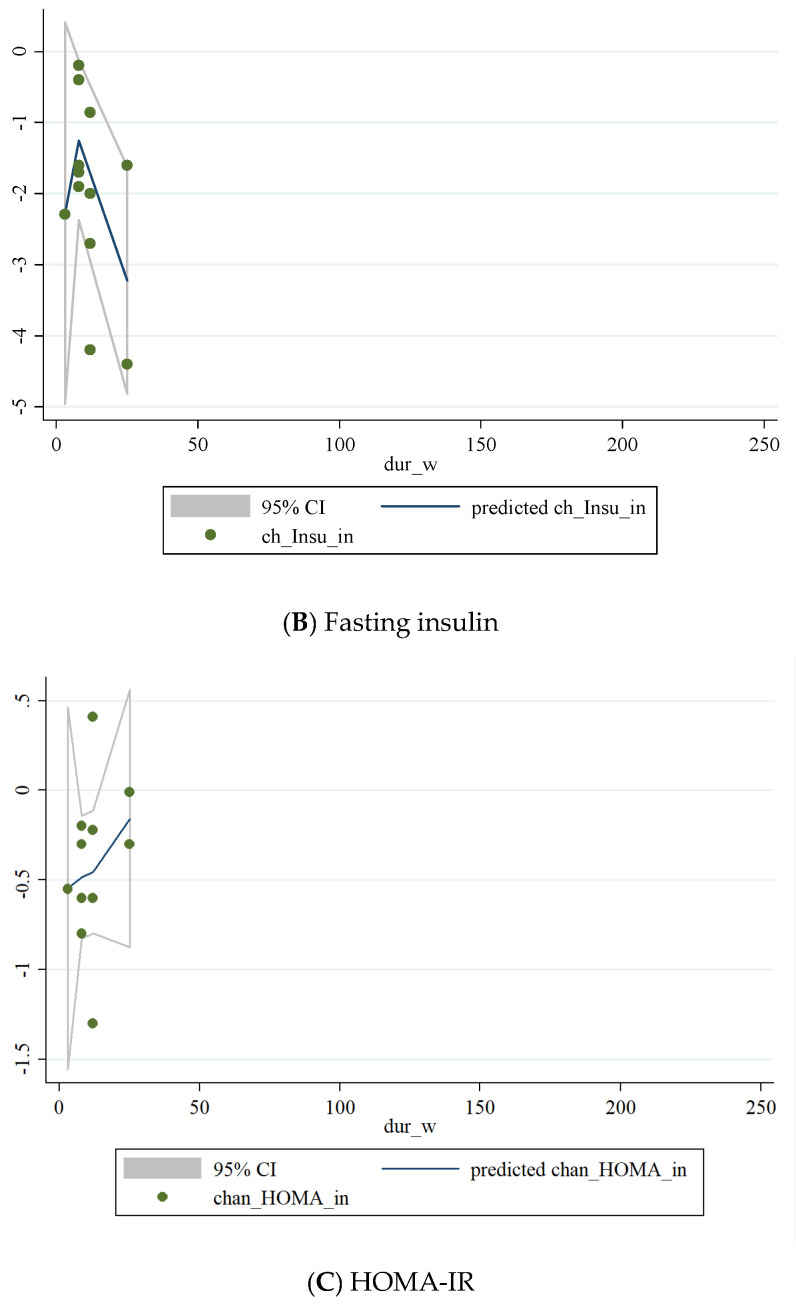
Non-linear dose-response relations between duration of intervention (weeks) and absolute mean differences in (**A**) FBG; (**B**) fasting insulin; (**C**) HOMA-IR.

We conducted a non-linear dose-response analysis between the dose and duration of folic acid supplementation for FBG, HOMA-IR, and fasting insulin concentrations. Dose-response analysis showed that folic acid supplementation significantly changed HOMA-IR (r = −1.30, *p*-nonlinearity = 0.045) in a non-linear fashion (Figure 4A–C). However, folic acid supplementation did not affect FBG, HOMA-IR, and fasting insulin concentrations based on the duration of the intervention (Figure 5A–C).

### 3.11. Meta-Regression Analysis

**Figure 6:**
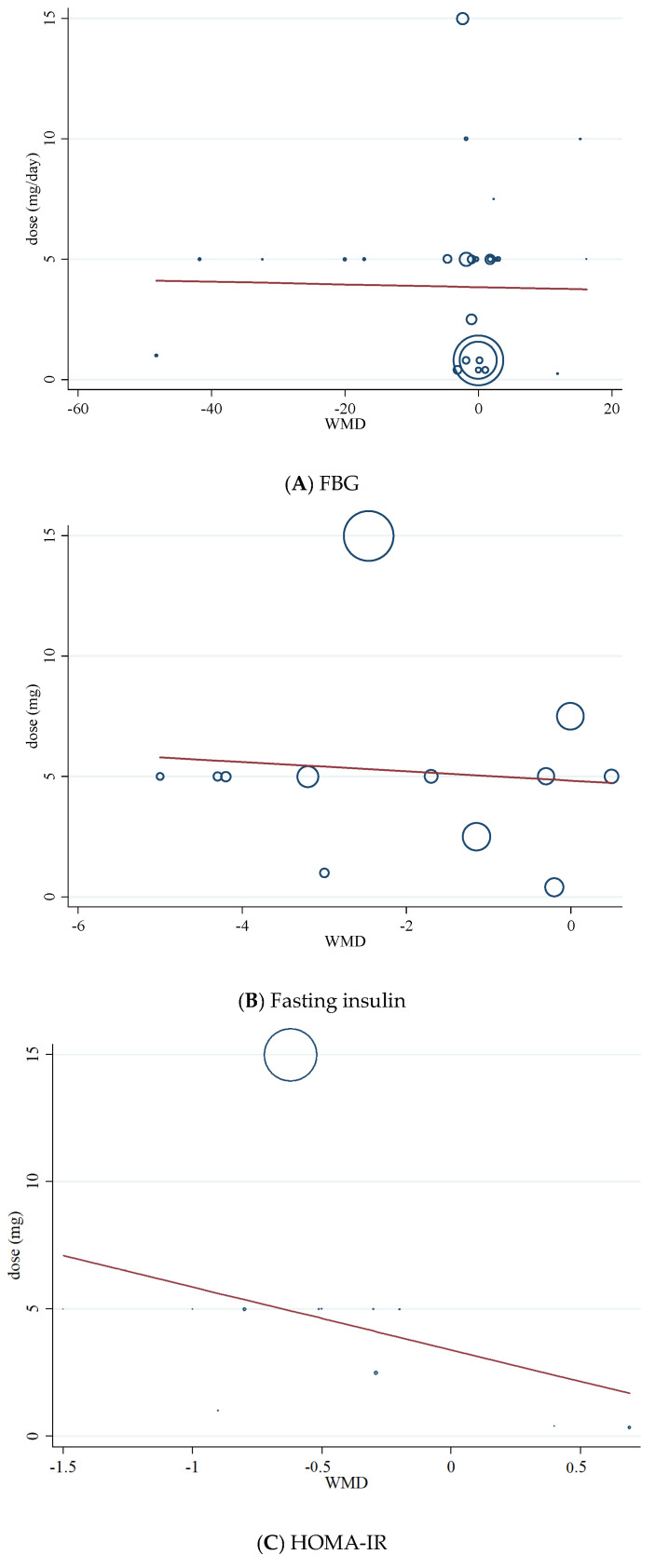
Random-effects meta-regression plots of the association between dose of folic acid (mg/day) and weighted mean difference of (**A**) FBG; (**B**) fasting insulin; (**C**) HOMA-IR.

**Figure 7:**
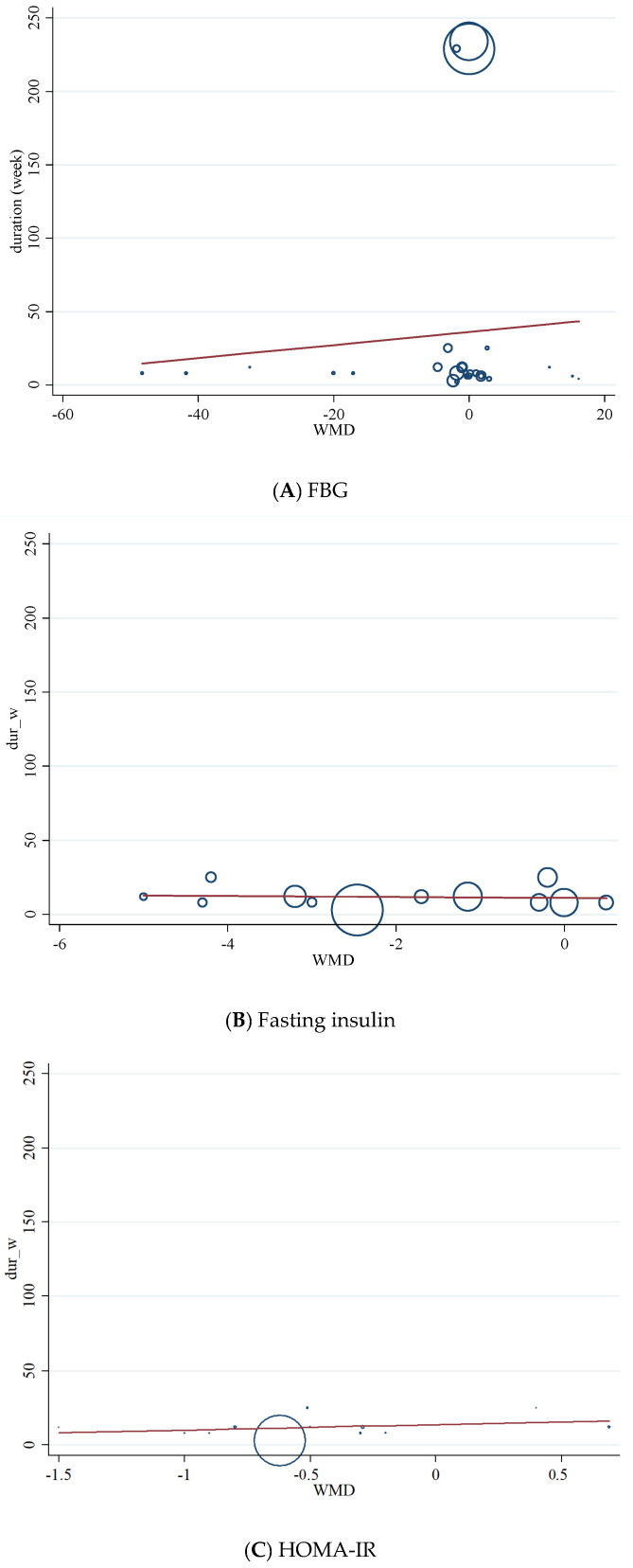
Random-effects meta-regression plots of the association between duration of intervention (weeks) and weighted mean difference of (**A**) FBG; (**B**) fasting insulin; (**C**) HOMA-IR.

Meta-regression was performed to investigate the potential association between a decrease in FBG, HOMA-IR, fasting insulin, dosage (mg/d), and duration (weeks) of folic acid supplementation. Meta-regression analysis did not indicate a linear relationship between dose (Figure 6A–C), duration (Figure 7A–C), and absolute changes in FBG, HOMA-IR, and fasting insulin concentrations.

## 4. Discussion

In this dose-response meta-analysis, we evaluated the effects of folic acid supplementation on glycemic control markers in adults. Results showed that folic acid supplementation reduced FBG, fasting insulin, and HOMA-IR, without any significant alterations in HbA1c when compared to a control group. Meanwhile, subgroup analyses on the dosage of folic acid supplementation (≥5 mg/d vs. <5 mg/d) showed that glycemic-improvement was more evident following higher doses. However, it should be noted that the improvements in measures of glycemic control following folic acid supplementation were relatively small and may not reach clinical importance.

Previous meta-analyses have reported inconsistent results regarding the efficacy of folic acid supplementation on various glycemic control markers, most notably FBG [23]. Akbari et al. examined the effects of folic acid supplementation in patients with metabolic diseases and found that folic acid supplementation resulted in significant decreases in insulin concentrations and HOMA-IR but did not affect FBG and HbA1c [23]. In another meta-analysis by Lind et al., folic acid supplementation lowered fasting insulin concentrations and HOMA-IR, with no effects observed for FBG or HbA1c [24]. Our results show improvements in glycemic markers of FBG, fasting insulin, and HOMA-IR, which is in agreement with the meta-analysis performed by Zhao et al., who found beneficial effects of folic acid supplementation on insulin resistance, FBG, and insulin [25]. However, we expand on the results of Zhao et al. because we performed sub-analyses involving a dose-response. According to our dose-response analysis, folic acid supplementation significantly improves insulin resistance in a non-linear fashion. Based on this finding, increasing the dose of folic acid supplementation to 5 mg/day can significantly improve insulin resistance. These findings are in line with previous observational studies, which reported that higher folate status could be associated with insulin resistance in people with obesity [51] and non-diabetic cohorts [52]. It seems that these beneficial effects of folic acid supplementation might be mediated by a decline in homocysteine concentrations [53].

Our results also demonstrate that folic acid supplementation resulted in a greater decline in fasting insulin concentrations in women compared with men. The reason for this phenomenon is not clear; however, some evidence suggests that folate concentrations and dietary folate intakes are significantly lower in females than males, which is reported in observational studies [15,54]. Moreover, it seems that men require more folic acid intake than women to achieve the same blood folate concentrations, mainly because men have a larger lean mass [55]. In addition, men had a much higher prevalence of cigarette smoking and drinking than women, and generally, smokers had lower plasma and red blood cell folate concentrations than nonsmokers [56,57,58]. On the other hand, some previous studies reported the positive relationship between serum folate and estrogen concentrations and the potential effects of folic acid supplementation on increasing estrogen concentrations among women [59,60]. Estrogen might decrease insulin concentrations by some possible mechanisms, including its anti-inflammatory and antioxidant properties such as maintaining glucose homeostasis and decreasing body fat [61]. Further studies are needed to evaluate the gender-specific effects of folic acid supplementation on glycemic markers.

Even though our subgroup analysis showed that individuals with higher FBS (>100 mg/dL) had greater improvements in this glycemic marker compared to those with lower FBS (<100 mg/dL), it should be noted that the effects of folic acid supplementation in our study were mostly observed in non-diabetic individuals. One possible explanation for the more relevant outcomes in non-diabetics might be the low number of studies on cohorts with diabetes (only 6 of the 24 included studies). Moreover, these studies were mostly short-term (less than 2 months) and consequently the complete benefit of folic acid supplementation may not have been observed during this period.

Our results failed to show significant decreases in HbA1c following folic acid supplementation, despite improvements in FBG, fasting insulin, and HOMA-IR. RBC life span is a known determinant of HbA1C [62], and it has been reported to average 80–115 days [63,64]. The four included studies, which evaluated HbA1C changes following folic acid supplementation had durations ranging from 4 to 12 weeks. Consequently, a plausible reason for the insignificant changes in HbA1C is the short period of interventions. Long-term studies evaluating HbA1C levels after folic acid supplementation are warranted.

The positive effects of folic acid supplementation on glycemic control markers may be related to habitual folic acid intake. Global folate status statistics indicate that folate deficiency varies from 5% to 20% in different countries [65]. In addition, 11 surveys reported the prevalence of folate insufficiency, which was >40% in most countries [66]. These data suggest the hypothesis that replenishment of folic acid concentrations in those who suffer from folate deficiency and insufficiency may have more favorable effects for improving glycemic control markers compared to individuals with normal folate concentrations. Based on World Health Organization (WHO) guidelines, concentrations of 13.5–45.3 nmol/L (6–20 ng/mL) is considered a normal range and a serum folate concentrations of <13.4 nmol/L (or <5.9 ng/mL) is interpreted as a possible deficiency [67]. From all 23 included studies, 17 studies reported baseline folate concentrations [15,20,21,22,32,33,34,35,37,38,39,40,41,42,43,47] and the baseline concentrations of folate in 9 studies are considered as possibly deficient [15,20,21,22,32,38,40,42,43]. Moreover, the recommended dietary allowance (RDA) for folic acid is 400 μg/day for both adult men and women [68]. From six studies, which did not reported baseline folate concentrations, four studies reported dietary intake of folic acid [44,45,48,50], and in all four of these studies, the average dietary folate intake was less than 280 μg/day. Therefore, since the participants of most included studies had lower folate concentrations than normal or low dietary intakes, folic acid supplementation showed significant improvement in the glycemic profile. From a mechanistic perspective, oxidative stress also leads to the production of reactive oxygen species (ROS) [69]. It has been shown that oxidative stress has been linked to the development of insulin resistance, β-cell dysfunction, impaired glucose tolerance, and mitochondrial dysfunction [70]. Alternatively, it has been mentioned that folic acid supplementation has antioxidant properties [71]. ROS-induced insulin resistance is mediated by some mechanism, including c-Jun N-terminal protein kinase (JNK) activation, tumor necrosis factor-alpha (TNF-α) increment decrease glucose uptake. In vitro and in vivo studies showed that ascorbic acid supplementation could decrease both JNK activation [72,73] and TNF-α gene expression [74]. Thirdly, inflammation plays an important role in the development of insulin resistance [75,76]. Previous investigations have revealed that proinflammatory cytokines such as TNF-α and IL-6 could impair insulin signaling, which may cause insulin resistance [75,76]. Moreover, both in vitro and in vivo studies have shown that folic acid supplementation may inhibit the proinflammatory process by inhibiting NF-kB activation [77,78]. Finally, folate-deficiency-induced homocysteinemia may have resulted in glycemic disorders [79,80,81]. Even earlier stages of folate deficiency are expressed by rising homocysteine concentrations, which appear at higher folic acid intakes [65]. Further mechanistic studies are needed to evaluate the possible mechanisms of the effects of folic acid supplementation on glycemic control.

This meta-analysis contains some strengths and limitations. The main strength of this study is the relatively acceptable number of studies and high sample size. Another advantage is the lack of publication bias in the analysis. Furthermore, we performed a dose-response analysis to evaluate the association between pooled effect size, dosage (mg/day), and duration of folic acid supplementation. Regarding limitations, it should be mentioned that all but three trials lasted less than three months; therefore, our analysis is unable to show the long-term effects of folic acid supplementation on glycemic profile. Moreover, there was a notable heterogeneity in the results of the investigations included.

In conclusion, folic acid supplementation may improve glycemic profile by decreasing FBG, fasting insulin, and HOMA-IR without altering HbA1c. It should be noted that the glycemic control properties of folic acid supplementation were small and may not reach clinical importance.

## Figures and Tables

**Table 1:** Characteristic of included studies.

Studies	Country	Study Design	Participant	Sample Size and Sex	Sample Size		Trial Duration (Week)	Means Age		Means BMI		Intervention	
					IG	CG		IG	CG	IG	CG	Folic Acid Dose (mg/d)	Control Group
Aarsand et al., 1998	Norway	RA/DB/PC (parallel)	type 2 diabetes	28: 21M, 7F	14	14	12	56.7 ± 10.47	61.6 ± 9.35	29.2 ± 5.23	28.3 ± 4.11	0.25	PC
Doshi et al., 2001	United Kingdom	RA/PC (parallel)	Coronary artery disease	50: 44M, 6F	50	50	6	57 ± 8	57 ± 8	28.5 ± 4.4	28.5 ± 4.4	5	PC
Doshi et al., 2002	United Kingdom	RA/PC (crossover)	Coronary artery disease	33: 30M 3F	16	17	6	55 ± 7	56 ± 7	NR	NR	5	PC
Kilicdag et al., 2005	Turkey	RA (parallel)	Polycystic ovarian syndrome patients	31: 31F	17	14	12	24.94 ± 6.67	24.14 ± 6.92	28.58 ± 5.43	26.02 ± 5.98	0.348	No intervention
Mangoni et al., 2005	Australia	RA/DB/PC (parallel)	Type 2 diabetes	26: 14M,12F	13	13	4	55.3 ± 4.32	57.6 ± 4.68	30.5 ± 3.96	32.3 ± 4.68	5	PC
Sheu et al., 2005	Taiwan	RA/DB/PC (parallel)	Obese women	74: 74F	36	38	12	43 ± 12	40 ± 12.32	29.6 ± 3.6	29.3 ± 4.93	5	PC
Villa et al., 2005	Italy	RA/PC (parallel)	Postmenopausal	20: 20F	10	10	8	55.4 ± 6.95	53.1 ± 7.27	29.7 ± 4.74	26.91 ± 5.88	7.5	PC
Moat et al., 2006 (A)	USA	RA/DB/PC (parallel)	Coronary artery disease	59: 52M, 7F	30	15	6	61 ± 7	61 ± 7	28.5 ± 4.4	29.6 ± 4.1	0.4	PC
Moat et al., 2006 (B)	USA	RA/DB/PC (parallel)	Coronary artery disease	54: 46M, 8F	25	14	6	60 ± 7	61 ± 7	29.9 ± 4.4	29.6 ± 4.1	5	PC
Solini et al., 2006	Italy	RA/PC (parallel)	Overweight subjects	60: 19M, 41F	30	30	12	50 ± 7	49 ± 8	27.5 ± 0.6	27.4 ± 0.6	2.5	PC
Title et al., 2006	Canada	RA/DB/PC (crossover)	Type 2 diabetes	19: 9M,10F	19	19	2	54.5 ± 5.9	54.5 ± 5.9	NR	NR	10	PC
Moens et al., 2007	Belgium	RA/DB/PC (crossover)	Acute myocardial infarction	40: 35M, 5F	20	20	6	57 ± 11	56 ± 14	NR	NR	10	PC
Mao et al., 2008 (A)	China	RA/DB (parallel)	Mild to moderate primary hypertension	295: 120M, 175F	146	75	8	57.4 ± 10	57.3 ± 10	25.5 ± 3.3	25.7 ± 3.2	0.4	No intervention
Mao et al., 2008 (B)	China	RA/DB (parallel)	Mild to moderate primary hypertension	297: 126M, 171F	148	74	8	56.6 ± 9.6	57.3 ± 10	25.8 ± 3.6	25.7 ± 3.2	0.8	No intervention
Palomba et al., 2010	Italy	DB/PC (parallel)	Polycystic ovary syndrome	47: 47F	23	24	25	26.9 ± 3.1	26.4 ± 2.8	27.9 ± 2.6	28.1 ± 3.1	0.4	PC
Aghamohammadi khiavi et al., 2011	Iran	RA/DB/PC (parallel)	Type 2 diabetes mellitus	68: 68M	34	34	8	58.7 ± 7.2	55.6 ± 9.3	27.4 ± 3.2	27.8 ± 4	5	PC
Gargari et al., 2011	Iran	RA/DB/PC (parallel)	Overweight and obese men with type 2 diabetes	48: 48M	24	24	8	59.4 ± 7.6	57 ± 10.1	28.8 ± 2.7	28.5 ± 3.3	5	PC
Grigoletti et al., 2013	Brazil	RA/DB/PC (parallel)	HIV-infected individuals	30: 14M, 16F	15	15	4	45 ± 7.74	45 ± 7.74	23.9 ± 4.96	23.9 ± 3.11	5	PC
Asemi et al., 2014 (A)	Iran	RA/DB/PC (parallel)	Overweight women with polycystic ovary syndrome	81: 81F	27	14	8	24.3 ± 5.0	24.7 ± 5.0	27.2 ± 5.0	27.9 ± 4.7	1	PC
Asemi et al., 2014 (B)	Iran	RA/DB/PC (parallel)	Overweight women with polycystic ovary syndrome	81: 81F	27	13	8	25.1 ± 4.5	24.7 ± 5.0	29.3 ± 4.6	27.9 ± 4.7	5	PC
Cagnacci et al., 2009	Italy	RA/DB/PC (parallel)	Postmenopausal	30: 30F	15	15	3	55.8 ± 4.26	54.5 ± 4.64	26.3 ± 5.03	27.5 ± 5.03	15	PC
Asemi et al., 2016	Iran	RA/DB/PC (parallel)	Cervical intraepithelial neoplasia grade 1	58: 58F	29	29	25	36.8 ± 8.8	39.1 ± 9.1	28.2 ± 3.5	29.8 ± 6.4	5	PC
Hashemi et al., 2016	Iran	RA/TB/PC (parallel)	Pre-eclamptic patients	85: 85F	43	42	8	30.82 ± 4.08	31.2 ± 4.3	25.19 ± 2.53	24.63 ± 2.64	5	PC
Qin et al., 2016	China	RA/DB (parallel)	Hypertension	20,030: 8295M, 11,735F	10,014	10,016	234	59.9 ± 7.6	60 ± 7.5	24.9 ± 3.7	24.9 ± 3.7	0.8	No intervention
Talari et al., 2016	Iran	RA/DB/PC (parallel)	Metabolic syndrome	60: 26M, 34F	30	30	12	62.1 ± 9.6	65.4 ± 11.5	29.8 ± 3.8	29.8 ± 4.4	5	PC
Li et al., 2017 (A)	China	RA/DB (parallel)	Diabetics	1636: 585M, 1051F	800	836	229	60.1 ± 7.2	59.9 ± 7.3	26.3 ± 3.7	26.4 ± 3.5	0.8	No intervention
Li et al., 2017 (B)	China	RA/DB (parallel)	Nondiabetics	11,435: 4444M, 6991F	5711	5724	229	59.4 ± 7.5	59.4 ± 7.6	25.5 ± 3.5	25.5 ± 3.5	0.8	No intervention
Bahmani et al., 2018	Iran	RA/DB/PC (parallel)	Endometrial hyperplasia	60: 60F	30	30	12	44.4 ± 6.5	44.7 ± 3.1	30.7 ± 4.6	30.5 ± 3.8	5	PC

Abbreviations: CG, control group; DB, double-blinded; F, female; IG, intervention group; M, male; NR, not reported; PC, placebo-controlled; RA, randomized; TB, triple-blinded; the references Moat et al., 2006, Mao et al., 2008, and Asemi et al., 2014 have 2 separate arms due to differences in population or folic acid supplementation dose (A and B).

**Table 2:** Quality assessment.

Studies	Random Sequence Generation	Allocation Concealment	Selective Reporting	Other Sources of Bias	Blinding (Participants and Personnel)	Blinding (Outcome Assessment)	Incomplete Outcome Data	Overall
Aarsand et al., 1998	L	H	H	H	L	H	H	Fair
Doshi et al., 2001	L	H	H	H	H	H	L	Fair
Doshi et al., 2002	L	H	H	H	H	H	L	Fair
Kilicdag Doshi et al., 2005	L	H	H	H	H	H	L	Fair
Mangoni et al., 2005	L	H	H	H	L	H	H	Fair
Sheu et al., 2005	L	H	H	H	L	H	L	Good
Villa et al., 2005	L	H	H	H	H	H	L	Fair
Moat et al., 2006	L	H	H	H	L	H	L	Good
Solini et al., 2006	L	H	H	H	H	H	L	Fair
Title et al., 2006	L	H	H	H	L	H	L	Good
Moens et al., 2007	L	H	H	H	L	H	L	Good
Mao et al., 2008	L	H	H	H	L	H	L	Good
Cagnacci et al., 2009	L	H	H	H	L	H	L	Good
Palomba et al., 2010	L	H	H	H	L	H	L	Good
Gargari et al., 2011	L	H	L	H	L	H	L	Good
khiavi et al., 2011	L	H	L	H	L	H	L	Good
Grigoletti et al., 2013	L	H	H	H	L	H	L	Good
Asemi et al., 2014	L	H	H	H	L	H	L	Good
Asemi et al., 2016	L	H	H	H	L	H	L	Good
Hashemi et al., 2016	L	L	H	H	L	L	L	Good
Qin et al., 2016	L	H	H	H	L	H	L	Good
Talari et al., 2016	L	H	H	H	L	H	L	Good
Bahmani et al., 2018	L	H	H	H	L	H	L	Good

Abbreviations: L, low; H, high.

**Table 3:** Subgroup analyses of folic acid supplementation on glycemic control in adults.

	NO	Sample Size (Intervention/Control)	WMD (95%CI)	*p*-Value	Heterogeneity			
					P Heterogeneity	I^2^	P between Sub-Groups	Tau-Squared
Subgroup analyses of folic acid supplementation on FBG								
Overall effect	27	17,379/17,235	−2.17 (−3.69, −0.65)	**0.005**	<0.001	81.5%		7.4032
Baseline FBG (mg/dL)								
<100	16	6365/6182	−2.14 (−4.36, −0.06)	0.057	<0.001	85.7%	0.824	13.20
≥100	11	11,014/11,053	−4.06 (−7.83, −0.29)	**0.043**	<0.001	71.9%		17.26
Trial duration (week)								
<12	17	662/464	−5.32 (−9.11, −1.53)	**0.006**	<0.001	86.5%	0.026	41.15
≥12	10	16,717/16,771	−0.79 (−1.81, 0.22)	0.126	0.041	48.6%		0.74
Intervention dose (mg/d)								
<5	10	16,943/16,822	−1.40 (−3.23, 0.43)	0.135	<0.001	84.9%	0.006	4.53
≥5	17	436/413	−3.58 (−6.62, −0.54)	**0.021**	<0.001	78.3%		21.89
Diabetes status								
non-T2DM	20	10,764/10,571	−2.34 (−4.46, −0.22)	**0.030**	<0.001	83.7%	0.243	14.16
T2DM	7	6615/6664	−4.87 (−10.15, 0.39)	0.070	0.001	73.6%		24.12
Sex								
Both sexes	16	17,081/16,962	0.11 (−0.55, 0.77)	0.905	0.653	0.0%	<0.001	0.00
Female	9	240/215	−9.53 (−14.71, −4.35)	**0.001**	<0.001	90.8%		39.33
Male	2	58/58	−18.81 (−26.87, −10.74)	**<0.001**	0.729	0.0%		0.00
	Overall analyses of folic acid supplementation on HbA1c							
Overall effect	4	85/85	−0.27 (−0.73, 0.18)	0.246	0.007	74.9%		0.16
Subgroup analyses of folic acid supplementation on fasting insulin								
Overall effect	12	315/291	−1.63 (−2.53, −0.73)	**<0.001**	0.001	65.8%		1.3281
Trial duration (week)								
<12	6	137/110	−1.28 (−2.73, 0.16)	0.082	0.001	76.0%	0.939	2.08
≥12	6	178/181	−2.03 (−3.31, −0.75)	**0.002**	0.045	55.8%		1.27
Intervention dose (mg/d)								
<5	3	80/68	−0.99 (−1.94, −0.04)	**0.040**	0.365	0.9%	0.082	0.00
≥5	9	235/223	−1.86 (−3.00, −0.71)	**0.001**	0.001	70.5%		1.76
Diabetes status								
non-T2DM	10	257/233	−1.96 (−2.92, −1.00)	**<0.001**	0.002	65.3%	0.015	1.22
T2DM	2	58/58	0.02 (−1.45, 1.51)	0.972	0.604	0.0%		0.00
Sex								
Both sexes	2	60/60	−2.37 (−5.89, 1.13)	0.185	0.119	58.8%	0.032	4.36
Female	8	197/173	−2.01 (−3.144, −0.88)	**<0.001**	0.002	69.0%		1.50
Male	2	58/58	0.02 (−1.45, 1.51)	0.972	0.604	0.0%		0.00
Subgroup analyses of folic acid supplementation on HOMA-IR								
Overall effect	12	322/295	−0.40 (−0.70, −0.09)	**0.011**	<0.001	80.9%		0.17
Trial duration (week)								
<12	5	127/100	−0.62 (−0.64, −0.59)	**<0.001**	0.654	0.0%	<0.001	0.00
≥12	7	195/195	−0.31 (−0.83, 0.19)	0.224	<0.001	83.7%		0.35
Intervention dose (mg/d)								
<5	4	97/82	0.02 (−0.68, 0.73)	0.949	<0.001	84.0%	<0.001	0.38
≥5	8	225/213	−0.62 (−0.64, −0.60)	**<0.001**	0.615	0.0%		0.00
Diabetes status								
non-T2DM	10	264/237	−0.43 (−0.77, −0.08)	**0.016**	<0.001	83.9%	0.192	0.19
T2DM	2	58/58	−0.26 (−0.79, 0.27)	0.339	0.857	0.0%		0.00
Sex								
Both sexes	2	58/58	−0.75 (−1.91, 0.39)	0.198	0.049	74.3%	0.103	0.54
Female	8	204/177	−0.38 (−0.82, 0.06)	0.092	<0.001	85.8%		0.28
Male	2	60/60	−0.26 (−0.79, 0.27)	0.339	0.857	0.0%		0.00

Abbreviations: CI, confidence interval; WMD, weighted mean differences; FBG, fasting blood glucose; HbA1c, hemoglobin A1c; HOMA-IR (homeostatic model assessment for insulin resistance); *p*-Value in bold: significant difference.

**Table 4:** GRADE profile of folic acid supplementation for FBG, HbA1c, fasting insulin and HOMA-IR scores in adults.

Quality Assessment						Summary of Findings		Quality of Evidence
Outcomes	Risk of Bias	Inconsistency	Indirectness	Imprecision	Publication Bias	Number of Intervention/Control	WMD (95%CI)	
FBG	No serious limitations	Very serious limitations ^a^	Serious limitations ^e^	No serious limitations	Serious limitations ^g^	17,379/17,235	−2.17 (−3.69, −0.65)	⊕◯◯◯ Very low
HbA1c	No serious limitations	Serious limitations ^b^	Serious limitations ^e^	Serious limitations ^f^	No serious limitations	85/85	−0.27 (−0.73, 0.18)	⊕◯◯◯ Very low
Fasting insulin	No serious limitations	Serious limitations ^c^	Serious limitations ^e^	No serious limitations	No serious limitations	315/291	−1.63 (−2.53, −0.73)	⊕⊕◯◯ Low
HOMA-IR	No serious limitations	Very serious limitations ^d^	Serious limitations ^e^	No serious limitations	No serious limitations	322/295	−0.40 (−0.70, −0.09)	⊕⊕◯◯ Low

^a^ The test for heterogeneity is significant, and the I^2^ is moderate, 83.2%. ^b^ The test for heterogeneity is significant, and the I^2^ is moderate, 74.9% ^c^ The test for heterogeneity is significant, and the I^2^ is moderate, 65.8%, ^d^ The test for heterogeneity is significant, and the I^2^ is moderate, 80.9%, ^e^ studies conducted subject to various conditions, ^f^ values are distributed within opposite direction across studies. ^g^ The Egger’s test for publication bias is significant (*p* = 0.039). Based on the number of limitations, the quality of outcomes is divided into four categories: high (⊕⊕⊕⊕), moderate (⊕⊕⊕◯), low (⊕⊕◯◯) and very low (⊕◯◯◯) quality.

## Data Availability

Data sharing is applicable with email to corresponding author.

## References

[1] Anioke I.C., Ezedigboh A.N., Dozie-Nwakile O.C., Chukwu I.J., Kalu P.N. (2019). Predictors of poor glycemic control in adult with type 2 diabetes in South-Eastern Nigeria. Afr. Health Sci..

[2] Razmpoosh E., Javadi A., Ejtahed H.S., Mirmiran P., Javadi M., Yousefinejad A. (2019). The effect of probiotic supplementation on glycemic control and lipid profile in patients with type 2 diabetes: A randomized placebo controlled trial. Diabetes Metab. Syndr. Clin. Res. Rev..

[3] Jalali M.T., Mohammadtaghvaei N., Larky D.A.J.B. (2016). Investigating the effects of Capparis spinosa on hepatic gluconeogenesis and lipid content in streptozotocin-induced diabetic rats. Biomed. Pharmacother..

[4] Afrisham R., Paknejad M., Soliemanifar O., Sadegh-Nejadi S., Meshkani R., Ashtary-Larky D. (2019). The influence of psychological stress on the initiation and progression of diabetes and cancer. Int. J. Endocrinol. Metab..

[5] Kooti W., Farokhipour M., Asadzadeh Z., Ashtary-Larky D., Asadi-Samani M.J. (2016). The role of medicinal plants in the treatment of diabetes: A systematic review. Electron. Physician.

[6] Abdi A., Mehrabani J., Nordvall M., Wong A., Fallah A., Bagheri R. (2020). Effects of concurrent training on irisin and fibronectin type-III domain containing 5 (FNDC5) expression in visceral adipose tissue in type-2 diabetic rats. Arch. Physiol. Biochem..

[7] Asbaghi O., Ashtary-Larky D., Bagheri R., Nazarian B., Pourmirzaei Olyaei H., Rezaei Kelishadi M., Nordvall M., Wong A., Dutheil F., Amirmansour Alavi N. (2021). Beneficial effects of folic acid supplementation on lipid markers in adults: A GRADE-assessed systematic review and dose-response meta-analysis of data from 21,787 participants in 34 randomized controlled trials. Crit. Rev. Food Sci. Nutr..

[8] Asbaghi O., Fatemeh N., Mahnaz R.K., Ehsan G., Elham E., Behzad N., Damoon N.L., Amirmansour A.N. (2020). Effects of chromium supplementation on glycemic control in patients with type 2 diabetes: A systematic review and meta-analysis of randomized controlled trials. Pharmacol. Res..

[9] Asbaghi O., Naeini F., Ashtary-Larky D., Moradi S., Zakeri N., Eslampour E., Kelishadim M.R., Naeini A.A. (2021). Effects of chromium supplementation on lipid profile in patients with type 2 diabetes: A systematic review and dose-response meta-analysis of randomized controlled trials. J. Trace Elem. Med. Biol..

[10] Asbaghi O., Ghanavati M., Ashtary-Larky D., Bagheri R., Rezaei Kelishadi M., Nazarian B., Nordvall M., Wong A., Dutheil F., Suzuki K. (2021). Effects of folic acid supplementation on oxidative stress markers: A systematic review and meta-analysis of randomized controlled trials. Antioxidants.

[11] Welch A.D. (1983). Folic acid: Discovery and the exciting first decade. Perspect. Biol. Med..

[12] Liew S.-C. (2016). Folic acid and diseases-supplement it or not?. Rev. Assoc. Médica Bras..

[13] Chen H., Liu S., Ji L., Wu T., Ji Y., Zhou Y., Zheng M., Zhang M., Xu W., Huang G. (2016). Folic acid supplementation mitigates Alzheimer’s disease by reducing inflammation: A randomized controlled trial. Mediat. Inflamm..

[14] Bahmani F., Karamali M., Shakeri H., Asemi Z. (2014). The effects of folate supplementation on inflammatory factors and biomarkers of oxidative stress in overweight and obese women with polycystic ovary syndrome: A randomized, double-blind, placebo-controlled clinical trial. Clin. Endocrinol..

[15] Gargari B.P., Aghamohammadi V., Aliasgharzadeh A. (2011). Effect of folic acid supplementation on biochemical indices in overweight and obese men with type 2 diabetes. Diabetes Res. Clin. Pract..

[16] Setola E., Monti L.D., Galluccio E., Palloshi A., Fragasso G., Paroni R., Magni F., Sandoli E.P., Lucotti P., Costa S. (2004). Insulin resistance and endothelial function are improved after folate and vitamin B12 therapy in patients with metabolic syndrome: Relationship between homocysteine levels and hyperinsulinemia. Eur. J. Endocrinol..

[17] Cagnacci A., Cannoletta M., Xholli A., Piacenti I., Palma F., Palmieri B. (2015). Folate administration decreases oxidative status and blood pressure in postmenopausal women. Eur. J. Nutr..

[18] Kurt R., Yilmaz Y., Ermis F., Besisik S.K., Polat N., Elitok A., Oflaz H., Karan M.A. (2010). Folic acid and vitamin B12 supplementation improves coronary flow reserve in elderly subjects with vitamin B12 deficiency. Arch. Med. Res..

[19] Chmurzynska A., Malinowska A.M., Twardowska-Rajewska J., Gawecki J. (2013). Elderly women: Homocysteine reduction by short-term folic acid supplementation resulting in increased glucose concentrations and affecting lipid metabolism (C677T MTHFR polymorphism). Nutrition.

[20] Mangoni A.A., Sherwood R.A., Asonganyi B., Swift C.G., Thomas S., Jackson S.H.D. (2005). Short-term oral folic acid supplementation enhances endothelial function in patients with type 2 diabetes. Am. J. Hypertens..

[21] Moens A.L., Claeys M.J., Wuyts F.L., Goovaerts I., Van Hertbruggen E., Wendelen L.C., Van Hoof V.O., Vrints C.J. (2007). Effect of folic acid on endothelial function following acute myocardial infarction. Am. J. Cardiol..

[22] Aarsand A., Carlsen S.J. (1998). Folate administration reduces circulating homocysteine levels in NIDDM patients on long-term metformin treatment. J. Intern. Med..

[23] Akbari M., Tabrizi R., Lankarani K.B., Heydari S.T., Karamali M., Kashanian M., Keneshlou F., Niknam K., Kolahdooz F., Asemi Z.J.H. (2018). The effects of folate supplementation on diabetes biomarkers among patients with metabolic diseases: A systematic review and meta-analysis of randomized controlled trials. Horm. Metab. Res..

[24] Lind M.V., Lauritzen L., Kristensen M., Ross A.B., Eriksen J.N. (2019). Effect of folate supplementation on insulin sensitivity and type 2 diabetes: A meta-analysis of randomized controlled trials. Am. J. Clin. Nutr..

[25] Zhao J.V., Schooling C.M., Zhao J.X. (2018). The effects of folate supplementation on glucose metabolism and risk of type 2 diabetes: A systematic review and meta-analysis of randomized controlled trials. Ann. Epidemiol..

[26] Moher D., Liberati A., Tetzlaff J., Altman D.G., Group P. (2009). Preferred reporting items for systematic reviews and meta-analyses: The PRISMA statement. PLoS Med..

[27] Higgins J.P., Altman D.G., Gøtzsche P.C., Jüni P., Moher D., Oxman A.D., Savović J., Schulz K.F., Weeks L., Sterne J.A. (2011). The Cochrane Collaboration’s tool for assessing risk of bias in randomised trials. BMJ.

[28] Asbaghi O., Sadeghian M., Nasiri M., Khodadost M., Shokri A., Panahande B., Pirouzi A., Sadeghi O. (2020). The effects of green coffee extract supplementation on glycemic indices and lipid profile in adults: A systematic review and dose-response meta-analysis of clinical trials. Nutr. J..

[29] Higgins J.P., Thompson S.G., Deeks J.J., Altman D.G. (2003). Measuring inconsistency in meta-analyses. BMJ.

[30] Tobias A. (1999). Assessing the influence of a single study in the meta-analysis estimate. Stata Tech. Bull..

[31] Gordon H., Oxman A., Vist G., Kunz R., Falck-Ytter Y., Alonso-Coello P., Schünemann H. (2008). Rating quality of evidence and strength of recommendations: GRADE: An emerging consensus on rating quality of evidence and strength of recommendations. BMJ.

[32] Doshi S.N., McDowell I.F., Moat S.J., Lang D., Newcombe R.G., Kredan M.B., Lewis M.J., Goodfellow J.J.A. (2001). Folate improves endothelial function in coronary artery disease: An effect mediated by reduction of intracellular superoxide?. Arterioscler. Thromb. Vasc. Biol..

[33] Doshi S.N., McDowell I.F., Moat S.J., Payne N., Durrant H.J., Lewis M.J., Goodfellow J.J.C. (2002). Folic acid improves endothelial function in coronary artery disease via mechanisms largely independent of homocysteine lowering. Circulation.

[34] Kilicdag E.B., Bagis T., Tarim E., Aslan E., Erkanli S., Simsek E., Haydardedeoglu B., Kuscu E.J.H.R. (2005). Administration of B-group vitamins reduces circulating homocysteine in polycystic ovarian syndrome patients treated with metformin: A randomized trial. Hum. Reprod..

[35] Sheu W.H.-H., Chin H.-M.L., Lee W.-J., Wan C.-J., Su H.-Y., Lang H.-F. (2005). Prospective evaluation of folic acid supplementation on plasma homocysteine concentrations during weight reduction: A randomized, double-blinded, placebo-controlled study in obese women. Life Sci..

[36] Villa P., Perri C., Suriano R., Cucinelli F., Panunzi S., Ranieri M., Mele C., Lanzone A. (2005). L-folic acid supplementation in healthy postmenopausal women: Effect on homocysteine and glycolipid metabolism. J. Clin. Endocrinol. Metab..

[37] Moat S.J., Madhavan A., Taylor S.Y., Payne N., Allen R., Stabler S.P., Goodfellow J., McDowell I., Lewis M.J., Lang D.J. (2006). High-but not low-dose folic acid improves endothelial function in coronary artery disease. Eur. J. Clin. Investig..

[38] Solini A., Santini E., Ferrannini E.J. (2006). Effect of short-term folic acid supplementation on insulin sensitivity and inflammatory markers in overweight subjects. Int. J. Obes..

[39] Title L.M., Ur E., Giddens K., McQueen M.J., Nassar B.A.J.V.M. (2006). Folic acid improves endothelial dysfunction in type 2 diabetes-an effect independent of homocysteine-lowering. Vasc. Med..

[40] Mao G., Hong X., Xing H., Liu P., Liu H., Yu Y., Zhang S., Jiang S., Wang X., Xu X.J.N. (2008). Efficacy of folic acid and enalapril combined therapy on reduction of blood pressure and plasma glucose: A multicenter, randomized, double-blind, parallel-controlled, clinical trial. Nutrition.

[41] Palomba S., Falbo A., Giallauria F., Russo T., Tolino A., Zullo F., Colao A., Orio F.J.D. (2010). Effects of metformin with or without supplementation with folate on homocysteine levels and vascular endothelium of women with polycystic ovary syndrome. Diabetes Care.

[42] Aghamohammadi Khiavi V., Pourghassem Gargari B., Aliasgharzadeh A. (2011). Effect of Folic Acid Supplementation on Indices of Glycemic Control, Insulin Resistance and Lipid Profile in Patients with Type 2 Diabetes Mellitus. IJEM.

[43] Grigoletti S.S., Guindani G., Moraes R.S., Ribeiro J.P., Sprinz E.J.N. (2013). Short-term folinic acid supplementation improves vascular reactivity in HIV-infected individuals: A randomized trial. Nutrition.

[44] Asemi Z., Karamali M., Esmaillzadeh A. (2014). Metabolic response to folate supplementation in overweight women with polycystic ovary syndrome: A randomized double-blind placebo-controlled clinical trial. Mol. Nutr. Food Res..

[45] Asemi Z., Vahedpoor Z., Jamilian M., Bahmani F., Esmaillzadeh A. (2016). Effects of long-term folate supplementation on metabolic status and regression of cervical intraepithelial neoplasia: A randomized, double-blind, placebo-controlled trial. Nutrition.

[46] Hashemi M., Heshmat-Ghahdarijani K., Zarean E., Baktash F., Mortazavi Z.S. (2016). Evaluation of the effect of high-dose folic acid on endothelial dysfunction in pre-eclamptic patients: A randomized clinical trial. J. Res. Med. Sci..

[47] Qin X., Li J., Zhang Y., Chen D., Wang B., He M., Fu J., Tang G., Cai Y., Shi X. (2016). Effect of folic acid supplementation on risk of new-onset diabetes in adults with hypertension in China: Findings from the China Stroke Primary Prevention Trial (CSPPT). J. Diabetes.

[48] Talari H.R., Rafiee M., Farrokhian A., Raygan F., Bahmani F., Mofrad M.D., Hamidian Y., Tamtaji O.R., Karamali F., Asemi Z. (2016). The Effects of Folate Supplementation on Carotid Intima-Media Thickness and Metabolic Status in Patients with Metabolic Syndrome. Ann. Nutr. Metab..

[49] Li Y., Liang M., Wang G., Wang B., He M., Tang G., Yin D., Xu X., Huo Y., Cui Y. (2017). Effects of folic acid therapy on the new-onset proteinuria in Chinese hypertensive patients: A post hoc analysis of the renal substudy of CSPPT (China Stroke Primary Prevention Trial). Hypertension.

[50] Bahmani F., Galougahi F.R., Vahedpoor Z., Jamilian M., Mahmoodi S., Baghban R., Bagherian T., Mehrizi M.Z., Asemi Z. (2018). The effects of folic acid supplementation on recurrence and metabolic status in endometrial hyperplasia: A randomized, double-blind, placebo-controlled trial. Arch. Iran. Med..

[51] Li Z., Gueant-Rodriguez R.-M., Quilliot D., Sirveaux M.-A., Meyre D., Gueant J.-L., Brunaud L. (2018). Folate and vitamin B12 status is associated with insulin resistance and metabolic syndrome in morbid obesity. Clin. Nutr..

[52] Li J., Goh C.E., Demmer R.T., Whitcomb B.W., Du P., Liu Z. (2017). Association between serum folate and insulin resistance among US nondiabetic adults. Sci. Rep..

[53] Kahleová R., Palyzová D., Zvára K., Zvárová J., Hrach K., Nováková I., Hyánek J., Bendlová B., Kožich V. (2002). Essential hypertension in adolescents: Association with insulin resistance and with metabolism of homocysteine and vitamins. Am. J. Hypertens..

[54] Robinson K., Arheart K., Refsum H., Brattström L., Boers G., Ueland P., Rubba P., Palma-Reis R., Meleady R., Daly L.J.C. (1998). Low circulating folate and vitamin B6 concentrations: Risk factors for stroke, peripheral vascular disease, and coronary artery disease. Circulation.

[55] Winkels R.M., Brouwer I.A., Verhoef P., Van Oort F.V., Durga J., Katan M. (2008). Gender and body size affect the response of erythrocyte folate to folic acid treatment. J. Nutr..

[56] Cafolla A., Dragoni F., Girelli G., Tosti M.E., Costante A., Pastorelli D., Bedogni G., Scott S.J.H. (2000). Folate status in Italian blood donors: Relation to gender and smoking. Haematologica.

[57] Bauer T., Göhlmann S., Sinning M.J.H.E. (2007). Gender differences in smoking behavior. Health Econ..

[58] Wilsnack R.W., Wilsnack S.C., Kristjanson A.F., Vogeltanz-Holm N.D., Gmel G.J.A. (2009). Gender and alcohol consumption: Patterns from the multinational GENACIS project. Addiction.

[59] Ericson U., Borgquist S., Ivarsson M.I., Sonestedt E., Gullberg B., Carlson J., Olsson H., Jirström K., Wirfält E. (2010). Plasma folate concentrations are positively associated with risk of estrogen receptor β negative breast cancer in a Swedish nested case control study. J. Nutr..

[60] Sütterlin M.W., Bussen S.S., Rieger L., Dietl J., Steck T. (2003). Serum folate and Vitamin B12 levels in women using modern oral contraceptives (OC) containing 20 μg ethinyl estradiol. Eur. J. Obstet. Gynecol. Reprod. Biol..

[61] Geer E.B., Shen W. (2009). Gender differences in insulin resistance, body composition, and energy balance. Gend. Med..

[62] Cohen R.M., Franco R.S., Khera P.K., Smith E.P., Lindsell C.J., Ciraolo P.J., Palascak M.B., Joiner C. (2008). Red cell life span heterogeneity in hematologically normal people is sufficient to alter HbA1c. J. Am. Soc. Hematol..

[63] Beltran del Rio M., Tiwari M., Amodu L.I., Cagliani J., Rodriguez Rilo H.L.R. (2016). Glycated hemoglobin, plasma glucose, and erythrocyte aging. J. Diabetes Sci. Technol..

[64] Franco R.S. (2012). Measurement of red cell lifespan and aging. Transfus. Med. Hemotherapy.

[65] Rogers L.M., Cordero A.M., Pfeiffer C.M., Hausman D.B., Tsang B.L., De-Regil L.M., Rosenthal J., Razzaghi H., Wong E.C., Weakland A. (2018). Global folate status in women of reproductive age: A systematic review with emphasis on methodological issues. Ann. N. Y. Acad. Sci..

[66] Eshak E.S., Iso H., Muraki I., Tamakoshi A. (2019). Among the water-soluble vitamins, dietary intakes of vitamins C, B-2 and folate are associated with the reduced risk of diabetes in Japanese women but not men. Br. J. Nutr..

[67] World Health Organization (2015). Serum and Red Blood Cell Folate Concentrations for Assessing Folate Status in Populations.

[68] Meyers L.D., Hellwig J.P., Otten J.J. (2006). Dietary Reference Intakes: The Essential Guide to Nutrient Requirements.

[69] Babel R.A., Dandekar M. (2020). A Review on Cellular and Molecular Mechanisms Linked to the Development of Diabetes Complications. Curr. Diabetes Rev..

[70] Rains J.L., Jain S.K. (2011). Oxidative stress, insulin signaling, and diabetes. Free. Radic. Biol. Med..

[71] Joshi R., Adhikari S., Patro B., Chattopadhyay S., Mukherjee T.J. (2001). Free radical scavenging behavior of folic acid: Evidence for possible antioxidant activity. Free. Radic. Biol. Med..

[72] Cianciulli A., Salvatore R., Porro C., Trotta T., Panaro M. (2016). Folic acid is able to polarize the inflammatory response in LPS activated microglia by regulating multiple signaling pathways. Mediat. Inflamm..

[73] Jiang M., Fan J., Qu X., Li S., Nilsson S.K., Sun Y.B.Y., Chen Y., Yu D., Liu D., Liu B.-C. (2019). Combined blockade of Smad3 and JNK pathways ameliorates progressive fibrosis in folic acid nephropathy. Front. Pharmacol..

[74] Samblas M., Martínez J.A., Milagro F. (2018). Folic acid improves the inflammatory response in LPS-activated THP-1 macrophages. Mediat. Inflamm..

[75] Shah A., Mehta N., Reilly M. (2008). Adipose inflammation, insulin resistance, and cardiovascular disease. J. Parenter. Enter. Nutr..

[76] Ferroni P., Basili S., Falco A., Davì G. (2004). Inflammation, insulin resistance, and obesity. Curr. Atheroscler. Rep..

[77] Bagherieh M., Kheirollahi A., Zamani-Garmsiri F., Emamgholipour S., Meshkani R. (2021). Folic acid ameliorates palmitate-induced inflammation through decreasing homocysteine and inhibiting NF-κB pathway in HepG2 cells. Arch. Physiol. Biochem..

[78] Kumar D., Singla S.K., Puri V., Puri S. (2015). The restrained expression of NF-kB in renal tissue ameliorates folic acid induced acute kidney injury in mice. PLoS ONE.

[79] Mursleen M.T., Riaz S. (2017). Implication of homocysteine in diabetes and impact of folate and vitamin B12 in diabetic population. Diabetes Metab. Syndr. Clin. Res. Rev..

[80] Cho N.H., Lim S., Jang H.C., Park H.K., Metzger B. (2005). Elevated homocysteine as a risk factor for the development of diabetes in women with a previous history of gestational diabetes mellitus: A 4-year prospective study. Diabetes Care.

[81] Mutavdzin S.S., Djuric D.M. (2020). Homocysteine and Related B Vitamins in Pre-diabetes and Diabetes Mellitus. Biochemistry of Cardiovascular Dysfunction in Obesity.

